# Co-designed and co-delivered place-based community interventions to reduce inequity in early initiation of antenatal care: findings from the cluster randomised controlled community REACH trial

**DOI:** 10.1136/jech-2024-223248

**Published:** 2025-12-11

**Authors:** Angela Harden, Meg Wiggins, Lorna Sweeny, Mary Sawtell, Cathryn Salisbury, Thomas Hamborg, Sandra Eldridge, Lauren Greenberg, Rachael Maree Hunter, Ekaterina Bordea, Christine McCourt, Bethan Hatherall, Gail Findlay, Adrian Renton, Ruth Ajayi, Ceri Durham, Adewale Adeyemo, Belinda Harvey, Kade Mondeh, Logan VanLessen

**Affiliations:** 1 City St George’s, University of London, London, UK; 2 Barts Health NHS Trust, London, UK; 3 Social Research Institute, UCL, London, UK; 4 Pragmatic Clinical Trials Unit, Wolfson Institute of Population Health, Queen Mary University of London, London, UK; 5 Wolfson Institute of Population Health, Queen Mary University of London, London, UK; 6 Region Hovedstaden, Hillerod, Denmark; 7 Department of Primary Care and Population Health, UCL, London, UK; 8 UCL, London, UK; 9 Centre for Maternal and Child Health, City St George’s, University of London, London, UK; 10 University of East London, London, UK; 11 Social Action for Health, London, UK; 12 North Middlesex University Hospital NHS Trust, London, UK; 13 Princess Alexandra Hospital NHS Trust, Harlow, UK; 14 Whittington Health NHS Trust, London, UK

**Keywords:** Health inequalities, HEALTH SERVICES, PREGNANCY

## Abstract

**Background:**

Early initiation of antenatal care provides timely screening, advice and support. Inequities in early care initiation exist in high-income countries, but there is scant evidence on effective interventions. The community REACH (Research for Equitable Antenatal Care and Health) trial aimed to assess the effectiveness of co-produced place-based interventions to strengthen community support for early care initiation.

**Methods:**

Matched-pair cluster randomised trial in socially disadvantaged and ethnically diverse areas in England. Electoral wards with low rates of early care initiation were matched and randomly allocated to intervention or control (usual care) (n=10 pairs). Following a 3-month co-design phase, community organisations and volunteers in intervention sites conducted targeted outreach activities over 6 months. The primary outcome was initiation of antenatal care by the 12th completed week of pregnancy.

**Results:**

There was no evidence of a difference in the primary outcome (OR 1.07, 95% CI 0.89 to 1.28). There were also no statistically significant differences in rates of emergency caesarean, preterm birth, low birth weight, smoking or breastfeeding. There was a higher rate of care initiation by 10 weeks and fewer antenatal admissions in the intervention arm during the intervention period, although differences were not sustained after it finished.

**Conclusion:**

This rigorous evaluation found a limited impact of short-term place-based interventions to strengthen community support for early initiation of antenatal care. Future initiatives may benefit from embedding in integrated health and care structures to ensure sufficient time and resources for mobilisation of community assets and focusing on smaller ‘hyper-local’ neighbourhoods. Actions to tackle wider structural and organisational barriers are also needed.

**Trial registration number:**

ISRCTN registry: registration number 63066975. Registered on 18 August 2015.

**WHAT IS ALREADY KNOWN ON THIS TOPIC**

- Previous research in high-income countries has identified inequalities in access to antenatal care, yet there is little evidence on interventions to improve early initiation of antenatal care.
- Co-produced place-based interventions which develop and strengthen community support offer a promising approach to tackle health inequalities, but this type of approach has not yet been rigorously tested in the context of early initiation of antenatal care in high-income countries.

**WHAT THIS STUDY ADDS**

- It was possible to develop and implement a co-designed and co-delivered place-based 6-month intervention to increase early access to antenatal care in ethnically and linguistically diverse inner-city neighbourhoods.
- There is no evidence that this short-term intervention increased the proportion of women accessing antenatal care before 12 completed weeks of pregnancy; initiation of care by 10 weeks increased significantly while the intervention was running, but this was not maintained after it ended.
- Intervention implementation varied across sites in the extent to which it reflected the underpinning intervention logic of co-production and community development; there was, however, no evidence of an association between implementation and intervention effect estimates.

**HOW THIS STUDY MIGHT AFFECT RESEARCH, PRACTICE OR POLICY**

- Given the results of this study, funders and intervention developers of future initiatives to strengthen community support should ensure a longer lead-in time so that interventions can be embedded within integrated health and care structures and allow sufficient time, resources and capability for co-production, the development of community relationships and asset-based ways of working.
- Efforts to tackle inequalities in early initiation of antenatal care should also consider service-level actions such as geographical location, availability of interpreters and anti-racist practice training.

## Introduction

 Reducing inequities in maternal and infant health outcomes is a priority for public health policy worldwide. In high-income countries such as the UK, socially disadvantaged groups and those from ethnically minoritised backgrounds continue to experience the worst outcomes.^1^ Antenatal care, the package of care provided from conception to the onset of labour, including components such as health promotion and screening, has been shown to prevent adverse outcomes, including maternal and neonatal mortality.^2^,^4^ Global and national policies recommend starting antenatal care within the first trimester for the early identification of pregnancy-related complications, health promotion and the development of trusting relationships with care givers,^2 5^ and late initiation of care has been associated with poorer outcomes.^1 4 6^ There are inequities in care initiation, with later access linked to socioeconomic deprivation and more likely among minoritised ethnic groups.^7^,^12^

Systematic reviews have found a dearth of evidence on what works to increase early initiation of antenatal care.^11 13 14^ Evidence on barriers and facilitators among those more likely to experience late access highlights the complexity of navigating health systems and a lack of promotion of the importance of early initiation of care.^9 13 15^ In high-income countries with universal free access to health services, these reviews suggest promising approaches are those in which maternity services collaborate with community organisations to promote early initiation in proactive, accessible and culturally safe ways. In low- and middle-income countries, participatory strategies involving peer leaders, advocacy and community health committees have been shown to promote earlier initiation of care.^16 17^

Place-based interventions which target the physical, economic or sociocultural aspects of place offer a promising approach for reducing health inequalities.^18 19^ Those that aim to change sociocultural aspects of a place (eg, community cohesion and support networks) fall into the ‘strengthening communities’ category of Whitehead’s typology of actions to reduce inequalities in health.^20^ It is imperative that marginalised and underserved communities are enabled to co-produce interventions that target them,^21^ and there is emerging evidence that when this happens, interventions are more likely to be effective.^22 23^ The effectiveness of co-produced place-based interventions which strengthen community support has not yet been rigorously tested regarding early initiation of antenatal care in high-income countries. This paper describes a study to address this gap.

The aims of this study’s intervention were to (1) raise awareness in local communities of the value of antenatal care and its early uptake and how to access it and (2) activate community assets and the wider health and care system to support early access. The longer-term aim was to change local social norms to sustain any increase in early initiation of antenatal care.

## Methods

### Trial design

Two-arm, matched-pair cluster randomised repeated cross-sectional trial, conducted in ethnically and linguistically diverse inner-city electoral wards in South East England.^24^ Electoral wards are used to elect local government councillors. While population size varies, the English average is about 8200 people per ward.^25^

The study included process and economic evaluations, the results of which are presented elsewhere.^26 27^ For the trial protocol, see Sawtell *et al*.^24^

### Intervention

**Figure 1:**
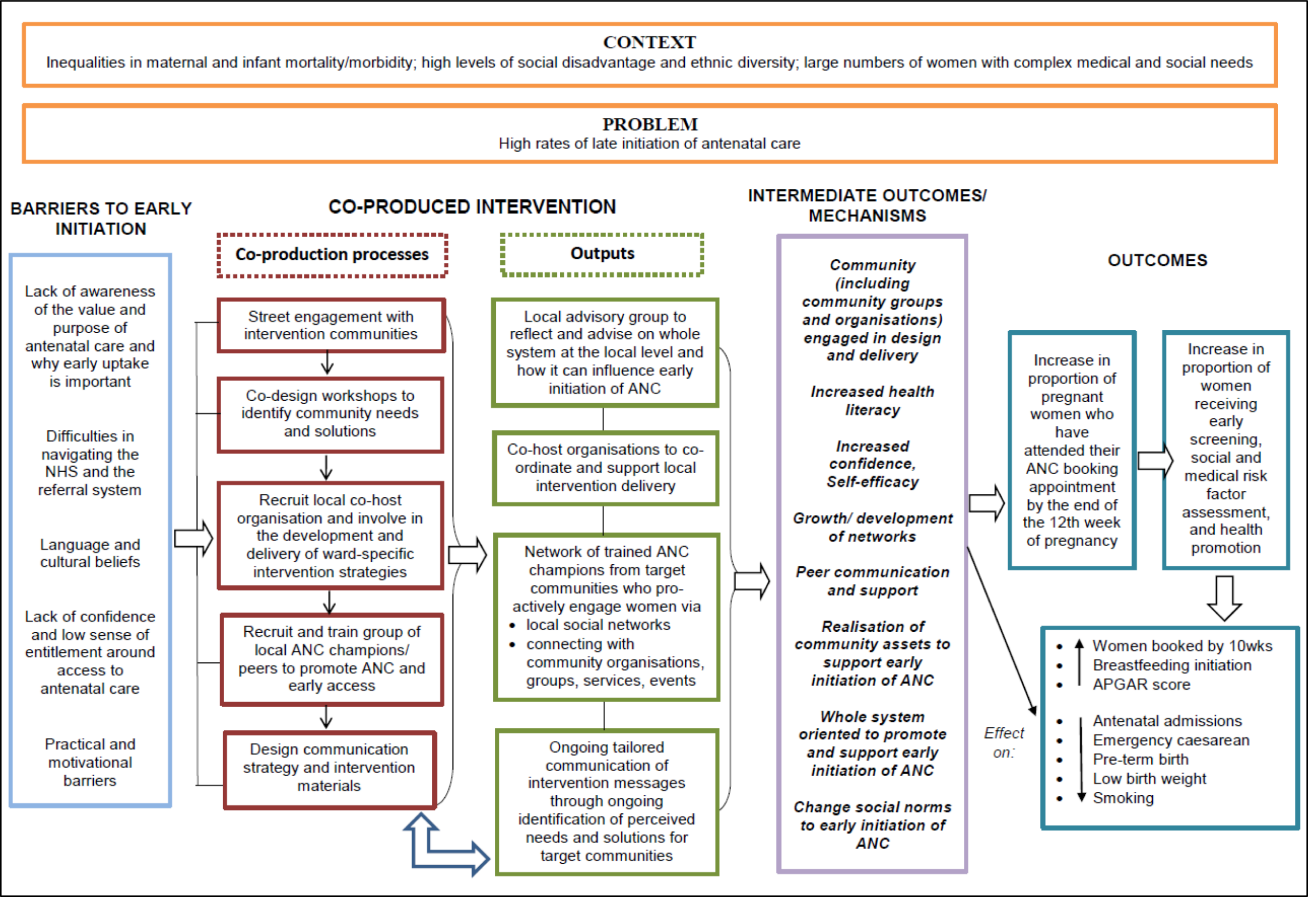
The Community REACH (Research for Equitable Antenatal Care and Health) intervention logic model (reproduced from Sawtell *et al*^24^). ANC, antenatal Care; APGAR, Appearance, Pulse, Grimace, Activity, and Respiration.

The intervention logic model was informed primarily by the concepts of co-production, community development and health literacy (figure 1). It was also informed by developmental work, including in-depth analysis of qualitative data, to examine barriers to early access among ethnically diverse and socially disadvantaged communities^7 9^ and the Well Communities framework for improving health in disadvantaged neighbourhoods.^28^ Well Communities specialists and a social design agency ran co-design workshops with residents and health and care system staff in each intervention site over a 3-month period. Intervention ideas were pooled and formed into an intervention plan with several components.

The intervention was directly targeted at whole communities and did not exclusively focus on women in the first trimester of pregnancy. In each area, a local community organisation managed the intervention over 9 months (3 months setup; 6 months intervention delivery). They convened local advisory groups to advise on key issues restricting early initiation of antenatal care and possible solutions. They recruited and trained community members as ‘antenatal champions’ to conduct conversations about the benefits of early care initiation and how to access care. These conversations were held with groups of people in their communities who were more likely to access care late, primarily women but also men. The intervention coordinator and antenatal champions also held targeted conversations with community leaders, local health and care services (eg, general practitioner practices) and community assets (eg, libraries, places of worship and local businesses such as barbershops) to generate community support for early care initiation. Further details are provided in Sawtell *et al*^24^ and in standardised format^29^ in online supplemental file 1.

### Recruitment and randomisation

Six National Health Service (NHS) Trusts were recruited between April and November 2015. The unit of randomisation was electoral wards served by these trusts. Wards with high delayed rates of initiation of antenatal care (<90% rate of antenatal booking by the end of the 12th completed week of pregnancy) were considered for inclusion into the study. The number of potential study wards was further reduced by removing wards so that no wards neighboured one another to reduce the likelihood of contamination. Ward pairs were matched on antenatal care initiation rates (low or very low), using data from a 6-month pretrial period. Within each pair, one ward was randomised to intervention and the other one to control (usual care), implying a 1:1 allocation ratio. Randomisation was conducted remotely by the Pragmatic Clinical Trials Unit at Queen Mary, University of London.

### Outcomes

The primary outcome was antenatal booking appointment attendance by the end of the 12th completed week of pregnancy (12 weeks and 6 days) as a binary (yes/no) variable. Secondary outcomes included attendance at antenatal booking appointment by 10 weeks+0 days of pregnancy, number of antenatal hospital admissions, smoking during pregnancy, proportion of emergency caesarean deliveries, proportion of preterm births, weight of baby at delivery, smoking at time of birth and infant feeding method at discharge from hospital.

### Data collection

All outcomes were collected via routine maternity data, provided electronically by data informatics teams from NHS Trusts participating in the study. In this repeated cross-sectional study design, outcome data were obtained from three different cohorts of women: cohort 1 (baseline) was prior to any intervention activities; cohort 2 (follow-up 1 (FU1)) was used to assess the treatment effect from 1 month after intervention commencement and cohort 3 (follow-up 2 (FU2)) was used to assess the sustained effect of the intervention after implementation ended. The period length for all three cohorts was 6 months, and data were collected for all births to women living within the study areas during these periods. Time periods determining cohorts for control sites were the same as for their paired intervention site. Follow-up data were extracted for the period June 2017 to October 2019.

### Sample size

The trial was designed to detect an increase in antenatal booking by 12 weeks and 6 days of gestation from 73% to 80%, with 90% power at the 5% significance level, accounting for clustering by ward (intracluster correlation coefficient=0.005) and assuming a mean cluster size of 130 and matching correlation=0.3. According to this sample size calculation, nine clusters in both intervention and control groups are required, which equates to at least 798 women per trial arm. To guard against loss of power if a cluster was lost, one cluster was added to each group, leading to a total recruitment target of 20 clusters.

### Statistical analysis

#### Main analysis

The primary outcome was analysed using a two-stage individual participant data meta-analysis technique for analysing paired cluster randomised controlled trials. Each matched pair of sites was regarded as an individual study in a meta-analysis for which the OR was estimated. These ORs were subsequently combined using a random effects model. Restricted maximum likelihood estimation was used. The Hartung-Knapp modification to the DeSimonian-Laird estimator of the between-study variance was used to construct t-based 95% CIs for effect estimators. All secondary outcomes were analysed using the same principles and analysis approach as the primary outcome, except for the outcome ‘number of antenatal admissions’. The use of the primary outcome model for count variables has not been described in the literature. This outcome was therefore analysed using a paired t-test on the mean number of admissions per site.

The first appearance/appointment of a woman in hospital within the routine maternity hospital data, up until and including birth, is used as the primary outcome value. The assumption was therefore that the amount of missing data would be negligible (<2%), and a blinded interim assessment of missingness confirmed this. The main analysis is therefore conducted on the available data without imputation.

#### Subgroup analysis

Subgroup analyses were planned on predefined subgroups (first vs subsequent pregnancy, ethnicity, deprivation (measured by Index of Multiple Deprivation (IMD)^30^), baseline booking rate and intervention model) for hypothesis generation, recognising that the study has limited power to detect differences at the subgroup level. All subgroup analyses were performed by estimating the treatment–covariate interaction within each pair of sites and pooling of the effect estimates thereafter.

Process data were used to assess intervention implementation.^26^ Each intervention site was assessed for (1) how closely implementation aligned with the community development ethos reflected in the logic model (scored high/med/low) and (2) the number of reported conversations between antenatal champions and targeted community members (high/med/low). This led to the construction of two models of implementation, which were used in subgroup analysis:

- *Model A*: sites where implementation had more focus on the number of conversations with community members and less focus on wider/more embedded community development.
- *Model B*: sites where implementation was more concentrated on embedded community development, but fewer conversations were reported.

#### Sensitivity analyses

The following analyses were carried out.

1. The treatment effect was estimated leaving out any site in which the intervention was not fully delivered as intended (per protocol analysis).
2. The treatment effect was estimated for the primary outcome using a model adjusted for individual-level covariates, IMD and ethnicity as fixed effects. Adjusted marginal proportion for treatment groups was estimated using the approach by Norton *et al*.^31^
3. Imputation of missing primary outcome values under different missing value assumptions.

#### Safety reporting

Maternal and infant deaths were extracted from hospital maternity records and analysed descriptively as safety measures across trial arms at each timepoint. Infant deaths included stillbirths as well as neonatal deaths.

The full statistical analysis plan, with dates of recruitment and follow-up, is publicly available at https://osf.io/cgfjw/.

### Patient and public involvement

Members of the public were involved in the trial in multiple ways. The trial was part of a programme grant which included two lay co-investigators on the investigator group; they are also co-authors on this paper. Residents from intervention wards were involved in the co-design and delivery of the intervention, and local community organisations managed intervention delivery.

## Results

### Study flow and participation

**Figure 2:**
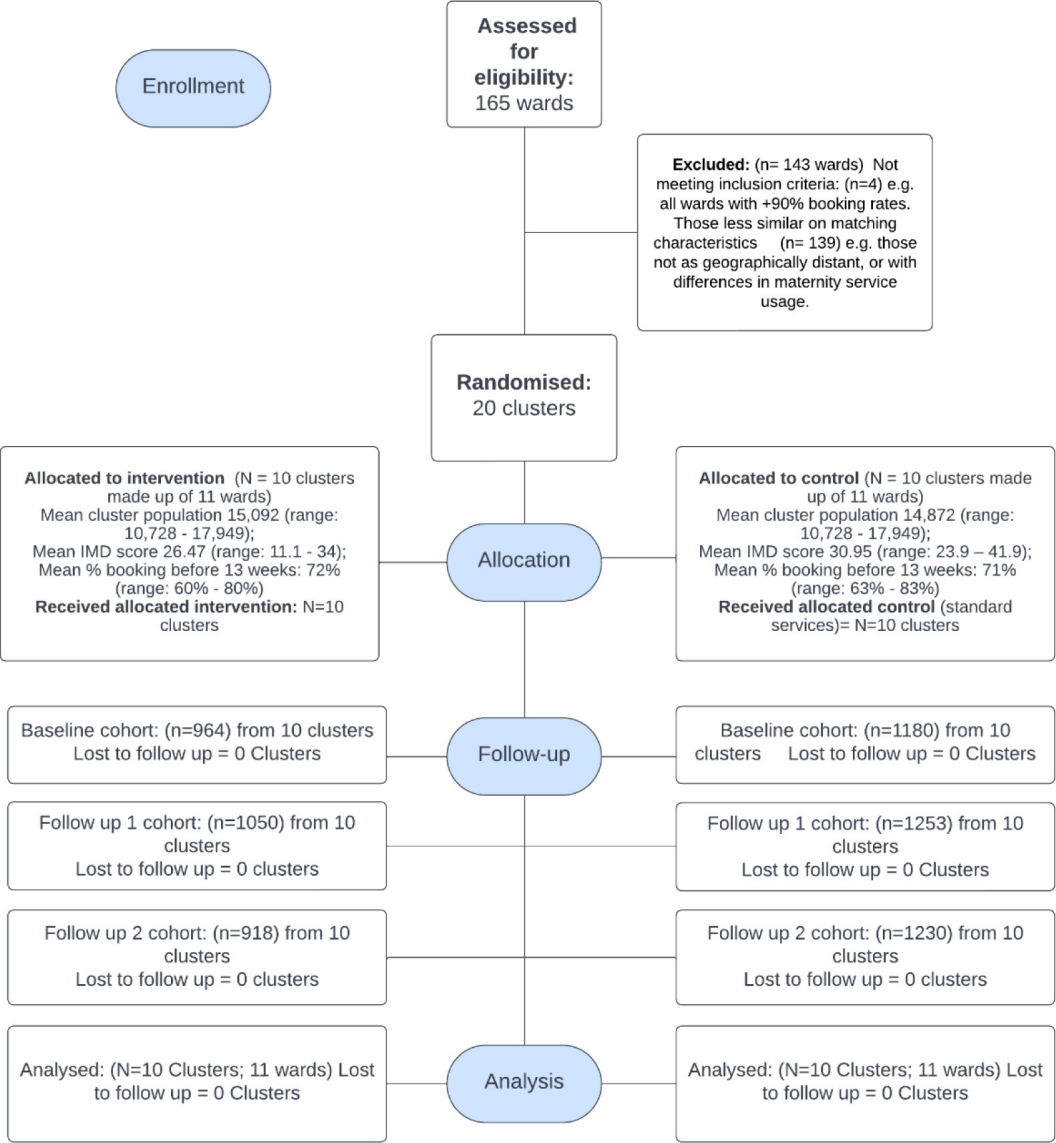
Community REACH trial consort diagram. IMD, Index of Multiple Deprivation.

The consort diagram (figure 2) indicates that all 10 sites allocated to the intervention arm participated, with programmes delivered in nine sites for the full intervention period of 6 months; in one site, delivery was for 3 months due to intervention set-up delays.

Data from the three time periods were provided for all intervention and control sites, and analysis was conducted with data from all sites.

### Balance between intervention and control sites

There was a relatively even balance between wards allocated to the two arms in terms of population size and booking rates before 13 weeks of pregnancy at baseline (figure 2). The wards within control sites were more deprived, with higher mean IMD scores than those within intervention sites (figure 2). Demographic data for the three cohorts also flagged that more of those in control sites lived in areas of greater deprivation. Other demographics were evenly balanced, with, for example, a high proportion of women from minoritised ethnic groups in both arms (table 1).

**Table 1:** Baseline characteristics

	Baseline cohort		First follow-up cohort		Second follow-up cohort	
	Intervention (n=964)	Control (n=1180)	Intervention (n=1050)	Control (n=1253)	Intervention (n=918)	Control (n=1230)
Women’s age at booking, N (%)						
<20	10 (1.34%)	33 (3.36%)	29 (3.32%)	31 (2.80%)	18 (1.96%)	42 (3.41%)
20–35	573 (76.71%)	770 (78.49%)	667 (76.32%)	843 (76.01%)	700 (76.25%)	930 (75.61%)
>35	164 (21.95%)	178 (18.14%)	178 (20.37%)	235 (21.19%)	200 (21.79%)	258 (20.98%)
Missing	217 (22.51%)	199 (16.86%)	176 (16.76%)	144 (11.49%)	0 (0.00%)	0 (0.00%)
Parity at booking, N (%)						
0	312 (36.19%)	404 (37.86%)	264 (30.70%)	344 (33.14%)	218 (28.24%)	316 (29.51%)
1–2	425 (49.30%)	486 (45.55%)	472 (54.88%)	497 (47.88%)	453 (58.68%)	550 (51.35%)
3+	125 (14.50%)	177 (16.59%)	124 (14.42%)	197 (18.98%)	101 (13.08%)	205 (19.14%)
Missing	102 (10.58%)	113 (9.58%)	190 (18.10%)	215 (17.16%)	146 (15.90%)	159 (12.93%)
Ethnicity, N (%)						
White British	180 (17.34%)	171 (12.89%)	177 (16.43%)	188 (13.83%)	182 (18.24%)	184 (14.06%)
White (other)	209 (20.13%)	355 (26.75%)	219 (20.33%)	365 (26.86%)	250 (25.05%)	395 (30.18%)
Asian	134 (12.91%)	179 (13.49%)	175 (16.25%)	198 (14.57%)	218 (21.84%)	223 (17.04%)
Black	103 (9.92%)	177 (13.34%)	107 (9.94%)	190 (13.98%)	111 (11.12)	215 (16.42%)
Other	101 (9.73%)	117 (8.82%)	102 (9.47%)	115 (8.46%)	94 (9.42%)	118 (9.01%)
Declined/unknown	94 (9.06%)	129 (9.72%)	120 (11.14%)	159 (11.70%)	143 (14.33%)	174 (13.29%)
Missing	217 (22.51%)	199 (16.86%)	177 (16.86%)	144 (11.49%)	0 (0.00%)	0 (0.00%)
Speaks English, N (%)						
Yes	199 (51.96%)	266 (55.53%)	225 (46.49%)	304 (49.92%)	227 (39.48%)	325 (43.45%)
Missing	581 (60.27%)	701 (59.41%)	566 (53.90%)	644 (51.40%)	343 (37.36%)	482 (39.19%)
Deprivation Index of Multiple Deprivation quintile, N (%)						
1–2	371 (38.49%)	644 (54.58%)	402 (38.29%)	680 (54.27%)	373 (40.63%)	689 (56.02%)
3–4	430 (44.61%)	481 (40.76%)	487 (46.38%)	514 (41.02%)	415 (45.21%)	490 (39.84%)
5–6	93 (9.65%)	51 (4.32%)	93 (8.86%)	54 (4.31%)	80 (8.71%)	46 (3.74%)
7–8	40 (4.15%)	4 (0.34%)	45 (4.29%)	5 (0.40%)	28 (3.05%)	5 (0.41%)
9–10	30 (3.11%)	0 (0%)	23 (2.19%)	0 (0%)	22 (2.40%)	0 (0%)
Missing	0 (0.00%)	0 (0.00%)	0 (0.00%)	0 (0.00%)	0 (0.00%)	0 (0.00%)
ToC after 12 weeks+6 days, N (%)						
Yes	45 (4.46%)	117 (9.02%)	42 (3.85%)	106 (7.80%)	57 (5.85%)	85 (6.46%)
Missing	440 (43.61%)	426 (32.85%)	345 (31.59%)	343 (25.24%)	213 (21.85%)	210 (15.97%)
Smoking at booking, N (%)						
Yes	53 (7.24%)	69 (7.09%)	69 (8.23%)	85 (8.00%)	55 (6.46%)	100 (8.69%)
Missing	232 (24.07%)	207 (17.54%)	212 (20.19%)	190 (15.16%)	67 (7.30%)	79 (6.42%)
Risk category, N (%)						
Standard	396 (53.88%)	495 (50.98%)	408 (40.22%)	549 (50.27%)	454 (50.56%)	577 (47.69%)
Intermediate	268 (36.46%)	364 (37.49%)	341 (39.47%)	437 (40.02%)	356 (39.64%)	500 (41.32%)
Intensive	71 (9.66%)	112 (11.53%)	115 (13.31%)	106 (9.71%)	88 (9.80%)	133 (10.99%)
Missing	229 (23.76%)	209 (17.71%)	186 (17.71%)	161 (12.85%)	20 (2.18%)	20 (1.63%)

ToC, Transfer of care. Women/birthing people could be transferred in to the maternity services in the trial from another service external to the trial. Transfers in after 12+6 days were excluded from the analysis.

### Outcomes findings

#### Main results for primary and secondary outcomes for FU1 cohort,

There was no statistically significant difference between arms in the primary outcome (initiation of antenatal care by the end of the 12th completed week of pregnancy) in the FU1 cohort in which outcomes were measured during the active implementation of the 6-month intervention (I=83.06%, C=82.46%; OR 1.07, 95% CI 0.89 to 1.28, p=0.440) (table 2). There were also no significant differences by trial arm for five of the seven secondary outcomes. There were similar rates of emergency caesarean, preterm birth, low birth weight, smoking at discharge and breastfeeding initiation in both the intervention and control arms (table 2). There was, however, a higher rate of initiation of antenatal care by 10 weeks in the intervention arm (I=49.47%; C=40.14%; OR 1.68, 95% CI 1.03 to 2.75, p=0.041). The mean number of antenatal hospital admissions was also lower in the intervention arm (I=0.47, C=0.56; mean difference=−0.13, 95% CI −0.24 to −0.01, p=0.030). (table 2). Sensitivity analyses suggest that the intervention effect estimate of the primary outcome is robust (online supplemental file 2).

**Table 2:** Main results for primary and secondary outcomes

	Included in analysis		Summary measure		Treatment effect		
	Intervention N (% miss)	Control N (% miss)	Intervention N (%)	Control N (%)	OR (95% CI)	OR, P value	RD (95% CI)
**Follow-up 1 cohort**							
Primary outcome							
Proportion of women having first antenatal appointment within 12 weeks+6 days	1039 (1.05%)	1243 (0.80%)	863 (83.06%)	1025 (82.46%)	1.07 (0.89 to 1.28)	0.440	0.01 (−0.01 to 0.04)
Secondary outcomes							
Proportion of participants having first antenatal appointment within 10 weeks+0 days	1039 (1.05%)	1243 (0.80%)	514 (49.47%)	499 (40.14%)	1.68 (1.03 to 2.75)	0.041	0.10 (0.01 to 0.19)
No. of hospital admissions (mean, med (SD))	1012 (3.62%)	1212 (3.27%)	0.47, 0, (0.82)	0.56, 0 (1.00)	−0.13 (−0.24 to −0.01)*	0.030	NA
Preterm birth (<37 weeks) (‘no’)	859 (17.56%)	1003 (18.65%)	783 (91.15%)	917 (91.43%)	0.95 (0.73 to 1.24)	0.692	−0.00 (−0.02 to 0.02)
Emergency caesarean section (‘no’)	876 (15.93%)	1018 (17.44%)	714 (81.51%)	828 (81.34%)	0.99 (0.65 to 1.52)	0.966	0.00 (−0.06 to 0.06)
Low birth weight (‘no’)	648 (37.81%)	714 (42.09%)	603 (93.06%)	657 (92.02%)	1.11 (0.76 to 1.63)	0.523	0.01 (−0.01 to 0.04)
Smoking at discharge (‘no’)	755 (28.10%)	899 (28.25%)	701 (92.85%)	831 (92.44%)	1.23 (0.74 to 2.05)	0.383	0.01 (−0.02 to 0.05)
Initiated breastfeeding (‘yes’)	786 (25.14%)	934 (25.46%)	711 (90.46%)	835 (89.40%)	1.28 (0.79 to 2.08)	0.276	0.02 (−0.02 to 0.06)
**Follow-up 2 cohort**							
Primary outcome							
Proportion of women having first antenatal appointment within 12 weeks+6 days	904 (1.53%)	1209 (1.71%)	708 (78.32%)	955 (78.99%)	0.98 (0.74 to 1.30)	0.888	−0.00 (−0.05 to 0.04)
Secondary outcomes							
Proportion of participants having first antenatal appointment within 10 weeks+0 day	904 (1.53%)	1209 (1.71%)	408 (45.13%)	493 (40.78%)	1.23 (0.85 to 1.78)	0.230	0.04 (−0.03 to 0.11)
No. of hospital admissions (mean, med (SD))	885 (2.66%)	1185 (2.94%)	0.50, 0, (1.00)	0.55, 0 (0.86)	−0.02 (−0.20 to 0.17)*	0.835	NA
Preterm birth (<37 weeks) (‘no’)	737 (18.83%)	997 (18.14%)	689 (93.49%)	931 (93.38%)	1.02 (0.71 to 1.47)	0.894	0.00 (−0.02 to 0.02)
Emergency caesarean section (‘no’)	765 (15.75%)	1031 (15.35%)	616 (80.52%)	857 (83.12%)	0.85 (0.57 to 1.26)	0.365	−0.02 (−0.04 to 0.03)
Low birth weight (‘no’)	567 (37.56%)	719 (40.97%)	526 (92.77%)	672 (93.46%)	0.93 (0.54 to 1.59)	0.747	−0.01 (−0.03 to 0.03)
Smoking at discharge (‘no’)	644 (29.85%)	909 (26.10%)	605 (93.94%)	834 (91.75%)	1.46 (0.61 to 3.51)	0.328	0.03 (−0.5 to 0.11)
Initiated breastfeeding (‘yes’)	680 (25.93%)	931 (24.31%)	613 (90.15%)	829 (89.04%)	1.22 (0.81 to 1.84)	0.295	0.01 (−0.02 to 0.05)

* Treatment effect estimate is mean difference between groups rather than OR.

N/A, Not applicable.

#### Main results for primary and secondary outcomes for the FU2 cohort

Results followed a similar pattern in the FU2 cohort in which outcomes were measured 3 months after the intervention had ended (table 2). There was a similar rate of initiation of antenatal care by 12 weeks and 6 days of pregnancy in both the intervention and control arms, and there were no statistically significant differences by trial arm in any of the secondary outcomes.

#### Subgroup analysis

Prespecified subgroup analyses of the primary outcome were conducted for parity, ethnicity, deprivation, cluster baseline rate of booking and implementation model. There was no evidence that the effect of the intervention differed by any of the variables considered (table 3).

**Table 3:** Subgroup analysis of primary outcome

	Intervention N (% missing)	Control N (% missing)	OR (95% CI)	P value for interaction
Parity of mother*				0.982
0	264 (0%)	343 (0.29%)	1.20 (0.64 to 2.27)	n/a
1+	588 (1.34%)	686 (1.15%)	1.14 (0.88 to 1.47)	n/a
Baseline rate of booking				
<70%	405 (1.22%)	551 (1.08%)	1.12 (0.74 to 1.68)	n/a
≥70% and <90%	634 (0.94%)	692 (0.57%)	1.04 (0.78 to 1.37)	n/a
Ethnicity†				0.600
White British	169 (0%)	169 (0%)	1.24 (0.15 to 9.93)	n/a
White (other)	162 (1.38%)	239 (1.01%)	0.88 (0.52 to 1.49)	n/a
Asian	170 (0.58%)	190 (1.04%)	0.76 (0.27 to 2.12)	n/a
Black	101 (0%)	183 (0%)	0.93 (0.48 to 1.79)	n/a
Other	97 (3.00%)	106 (0.93%)	1.53 (0.77 to 3.04)	n/a
Deprivation Index of Multiple Deprivation quintile‡				0.310
1–2	77 (0%)	241 (0.82%)	1.17 (0.21 to 6.48)	n/a
3–4	323 (0.62%)	432 (1.14%)	1.03 (0.62 to 1.72)	n/a
5–6	364 (1.09%)	322 (0.92%)	0.83 (0.39 to 1.76)	n/a
7–8	114 (4.20%)	189 (0%)	0.99 (0.18 to 5.35)	n/a
9–10	78 (0%)	43 (0%)	1.85 (0.39 to 8.73)	n/a
Intervention implementation				0.595
*Model A* (more focus on quantity of conversations and less focus on community development)	701 (0.99%)	772 (1.03%)	1.09 (0.84 to 1.40)	n/a
*Model B* (less focus on quantity of conversations and more focus on community development)	338 (1.17%)	471 (0.42%)	1.00 (0.89 to 1.28)	n/a

* Only six ward pairs used.

† Only seven ward pairs used.

‡ Only four ward pairs used.

N/A, Not applicable.

#### Safety reporting

There were no maternal deaths during the study period. Infant death rates were similar between trial arms (see online supplemental file 3).

## Discussion

This study succeeded in developing and implementing co-produced place-based interventions to increase early antenatal care initiation in 10 ethnically and linguistically diverse inner-city neighbourhoods. This was rigorously evaluated through a large-scale cluster randomised trial. The intervention did not show differences between the trial arms at either of the two follow-up time points in the primary outcome of accessing antenatal care before 12 completed weeks of pregnancy. The secondary outcomes also showed limited differences, although the proportion of women who accessed care by 10 weeks was higher and antenatal admissions were lower in the intervention arm at the first follow-up while the intervention was running.

In considering why there was a limited effect, our process evaluation pointed to several issues.^26^ The interventions were dependent on the trickle-down of messaging through community assets as well as direct contact with those in the neighbourhood. A focus on the number of conversations by those commissioning and delivering the intervention meant that in some areas there was insufficient resource to put into fostering relationships with community assets, while in other areas the reverse applied. Running the intervention for longer than 6 months with bigger teams and including organisational development to support asset-based working may be needed to increase intervention strength for changing the sociocultural environment to support early access to antenatal care. Similarly, our intervention may not have sufficiently reached those who were most at risk of late initiation of care, despite using local information in each site to identify key target groups. Some previous research has suggested that antenatal care as currently provided is not always attractive to ethnically diverse and disadvantaged groups.^9^ As this intervention did not change the model of antenatal care provided, uncertainty remains whether increasing the length and reach of the intervention would have the impact indicated by the process evaluation.

The current staffing crisis in midwifery, combined with the austerity of healthcare budgets in the UK, has placed additional limits on maternity services. Our intervention may have created extra demand for earlier initiation of antenatal care, which services may not have been able to meet. Recent systematic reviews on engagement with antenatal care have pointed to the importance of addressing structural and organisational barriers for deeply entrenched health inequities. Recommendations from Sharma *et al*^15^ for structural adaptations for accessible antenatal services for ethnically minoritised groups included the geographical location of services, the availability of interpreters and antiracist and culturally inclusive practice training. These types of changes are important but were beyond the scope of our intervention.

A large-scale randomised controlled trial requires rigour, which was at times difficult to marry with the flexibility required in a co-produced community engagement intervention. Similar constraints have been expressed by others.^32^,^34^ We found that our unit of randomisation—electoral wards—set up artificial geographical boundaries, which did not always reflect the neighbourhood areas defined by the communities with which we engaged. Indeed, their social networks often transcended geographical boundaries. From a community development perspective, wards are relatively large geographical areas; in smaller ‘hyper-local’ neighbourhoods, community networks to stimulate change can be more easily developed.

Despite the limited positive results for this specific intervention, this trial does offer learning that those developing and implementing interventions to address health inequalities in health and care services can take forward regarding the co-production of place-based community interventions.^35^ In carrying out this work to strengthen community support for early care initiation, we brought together and helped forge relationships between the UK NHS and the wider community across 10 areas at a time when working was very siloed. We found, however, that the absence of structures in place for engagement meant these processes often had no prior foundation and required more time and resources to develop impactful relationships. Such findings tally with those of South and colleagues in their study of the community champion model, which rose in prominence at the height of the COVID-19 pandemic for its ability to connect with groups who were disproportionately impacted.^36 37^ They also noted that a supportive infrastructure, training in community engagement and development of long-term community relationships were required.

This is a time of system transformation in the UK NHS, with new Integrated Care Systems providing greater recognition and incentives for services to work with the community to reduce health inequalities.^38^ This, and similar policy drivers in other countries towards community asset-based ways of working, could create better opportunities for embedding place-based interventions to strengthen community support for addressing health inequalities in maternity care in the future.

## Supplementary material

10.1136/jech-2024-223248 online supplemental file 1

10.1136/jech-2024-223248 online supplemental file 2

10.1136/jech-2024-223248 online supplemental file 3

## Data Availability

Data are available upon reasonable request.
