# Supplementary material for: Co-designed and co-delivered place-based community interventions to reduce inequity in early initiation of antenatal care: findings from the cluster randomised controlled community REACH trial
Source: J Epidemiol Community Health. 2025 Dec 11;80(3):e223248. doi: 10.1136/jech-2024-223248 (PMC13018804; doi:10.1136/jech-2024-223248)
Supplement: online supplemental file 2 [file jech-80-3-s002.pdf]

Table S2: Sensitivity analysis of primary outcome

	Included in analysis		Treatment effect		
	**Intervention N (%)**	**Control N (%)**	**OR (95% CI)**	**OR p-value**	**RD (95% CI)**
Per protocol analysis	924 (88.0%)	1088 (86.8%)	1.05 (0.85; 1.29)	0.604	0.01 (-0.01; 0.04)
Adjusted for ethnicity and IMD	865 (82.4%)	1102 (87.9%)	1.08 (0.68; 1.72)	0.711	0.01 (-0.06; 0.08)
**Imputation of missing primary outcomes**					
Substitution of all missing values of the outcome as having booked after 12 weeks + 6 days for both arms.	1050 (100%)	1253 (100%)	1.06 (0.89; 1.26)	0.480	0.01 (-0.01; 0.04)
Substitution of all missing values of the outcome as having booked after 12 weeks + 6 days in the intervention arm and before 12 weeks + 6 days in control arm wards*.	1050 (100%)	1253 (100%)	0.99 (0.83; 1.20)	0.950	0.01 (-0.02; 0.03)
Substitution of all missing values of the outcome as having booked before or after 12 weeks + 6 days with probability 0.5 in both arms	1050 (100%)	1253 (100%)	1.06 (0.88; 1.28)	0.478	0.01 (-0.01; 0.04)

*This is the most extreme imputation option and provides a lower limit for potential effect estimates.
