# Supplementary material for: Co-designed and co-delivered place-based community interventions to reduce inequity in early initiation of antenatal care: findings from the cluster randomised controlled community REACH trial
Source: J Epidemiol Community Health. 2025 Dec 11;80(3):e223248. doi: 10.1136/jech-2024-223248 (PMC13018804; doi:10.1136/jech-2024-223248)
Supplement: online supplemental file 3 [file jech-80-3-s003.pdf]

**Supplementary file 3: Safety report**

| | Baseline | | Follow-up 1 | | Follow-up 2 | |
|---|---|---|---|---|---|---|
| | **Intervention (n=882)** | **Control (n=1089)** | **Intervention (n=969)** | **Control (n=1148)** | **Intervention (n=840)** | **Control (n=1143)** |
| Maternal death N(%) | 0 (0.00%) | 1 (0.09%) | 0 (0.00%) | 0 (0.00%) | 0 (0.00%) | 0 (0.00%) |
| Infant death N(%) | 3 (0.34%) | 3 (0.28%) | 3 (0.29%) | 9 (0.72%) | 2 (0.24%) | 3 (0.26%) |
